# miR-145 mediates the antiproliferative and gene regulatory effects of vitamin D3 by directly targeting E2F3 in gastric cancer cells

**DOI:** 10.18632/oncotarget.3048

**Published:** 2015-02-18

**Authors:** Su'e Chang, Ling Gao, Yang Yang, Dongdong Tong, Bo Guo, Liying Liu, Zongfang Li, Tusheng Song, Chen Huang

**Affiliations:** ^1^ Department of Genetics and Molecular Biology/Key Laboratory of Environment and Genes Related to Diseases, College of Medicine, Xi'an Jiaotong University, Xi'an, Shaanxi, P. R. China; ^2^ Department of Oral Maxillofacial Surgery, Stomatology Hospital of Xi'an Jiaotong University College of Medicine, Xi'an, Shaanxi, P. R. China; ^3^ Department of General Surgery, the Second Affiliated Hospital, School of Medicine, Xi'an Jiaotong University, Xi'an, Shaanxi, P. R. China

**Keywords:** 1,25(OH)_2_D_3_, miR-145, gastric cancer, E2F3, proliferation

## Abstract

VitaminD3 signaling is involved in inhibiting the development and progression of gastric cancer (GC), while the active vitamin D metabolite 1-alpha,25-dihydroxyvitamin D3 (1,25(OH)_2_D_3_)-mediated gene regulatory mechanisms in GC remain unclear. We found that miR-145 is induced by 1,25(OH)_2_D_3_ in a dose- and vitamin D receptor (VDR)-dependent manner in GC cells. Inhibition of miR-145 reverses the antiproliferative effect of 1,25(OH)_2_D_3_. Furthermore, miR-145 expression was lower in tumors compared with matched normal samples and correlated with increased the E2F3 transcription factor protein staining. Overexpression of miR-145 inhibited colony formation, cell viability and induced cell arrest in S-phase in GC cells by targeting E2F3 and CDK6. miR-145 inhibition consistently abrogates the 1,25(OH)_2_D_3_-mediated suppression of *E2F3*, *CDK6*, *CDK2* and *CCNA2* genes. Altogether, our results indicate that miR-145 mediates the antiproliferative and gene regulatory effects of vitamin D3 in GC cells and might hold promise for prognosis and therapeutic strategies for GC treatment.

## INTRODUCTION

Gastric cancer (GC) is currently the fourth most common malignancy in the world. It can spread throughout the stomach and to other organs, including the esophagus, lungs, lymph nodes or liver. While the etiology of GC carcinogenesis is thought to be multifactorial, molecular analysis coupled with genome-wide approaches has identified various genetic alterations related to gastric tumorigenesis and progression [1]. Recently, the correlation between vitamin D3 and microRNAs (miRNAs) has open new opportunities for cancer research, including GC research.

Epidemiology studies showed that vitamin D insufficiency or deficiency increases cancer incidence, particularly for cancers of the digestive system [2]. The most active vitamin D3 metabolite 1alpha,25-dihydroxyvitamin D3 (1,25(OH)_2_D_3_) affects cancer development and growth through regulating multiple signaling pathways involved in cell proliferation, apoptosis, invasion, and metastasis [3–5]. 1,25(OH)_2_D_3_ (also known as calcitriol) functions by binding to and activating nuclear vitaminD receptor (VDR), which is a ligand-modulated transcription factor that binds to specific sequences (vitamin D response elements (VDRE)) in target genes and increases or decreases their transcription rate through interaction with a vast array of co-activators, co-repressors, chromatin modifier enzymes, and remodeling complexes [6]. Thereby, nearly 3–5% of human genes are regulated by 1,25(OH)_2_D_3_ directly or indirectly, these include miRNAs, which play very important roles in cancer development [7–9].

miRNAs are known as master gene regulators by base pairing with mRNAs, leading to mRNA inhibition or destabilization. Deregulation of certain miRNAs may contribute to human cancer and miRNAs function as tumor suppressors or oncogenes in cancer cells [10]. Generally, miRNAs target multiple mRNAs. An individual miRNA could potentially alter complex cellular processes such as cell growth, cell cycle, apoptosis and invasion. Aberrant miRNA levels are observed in many tumors, and some deregulated miRNAs such as miR-338-3p, miR-204, miR-508-5p, miR-16, miR-21, miR-27a, and miR-19b/20a/92a have been identified in GC [1, 11–17].

Data suggest that serum vitamin D level is a significant independent prognostic factor in patients with GC, and that vitamin D deficiency may be associated with poor prognosis [18]. In this study, we show that miR-145 is deregulated in GC and induced by 1,25(OH)_2_D_3_. We aim to confirm and extend our findings through the identification of target miRNAs for 1,25(OH)_2_D_3_, and to analyze their biological mechanism in GC.

## RESULTS

### 1,25(OH)_2_D_3_ inhibits GC cell proliferation

**Figure 1:**
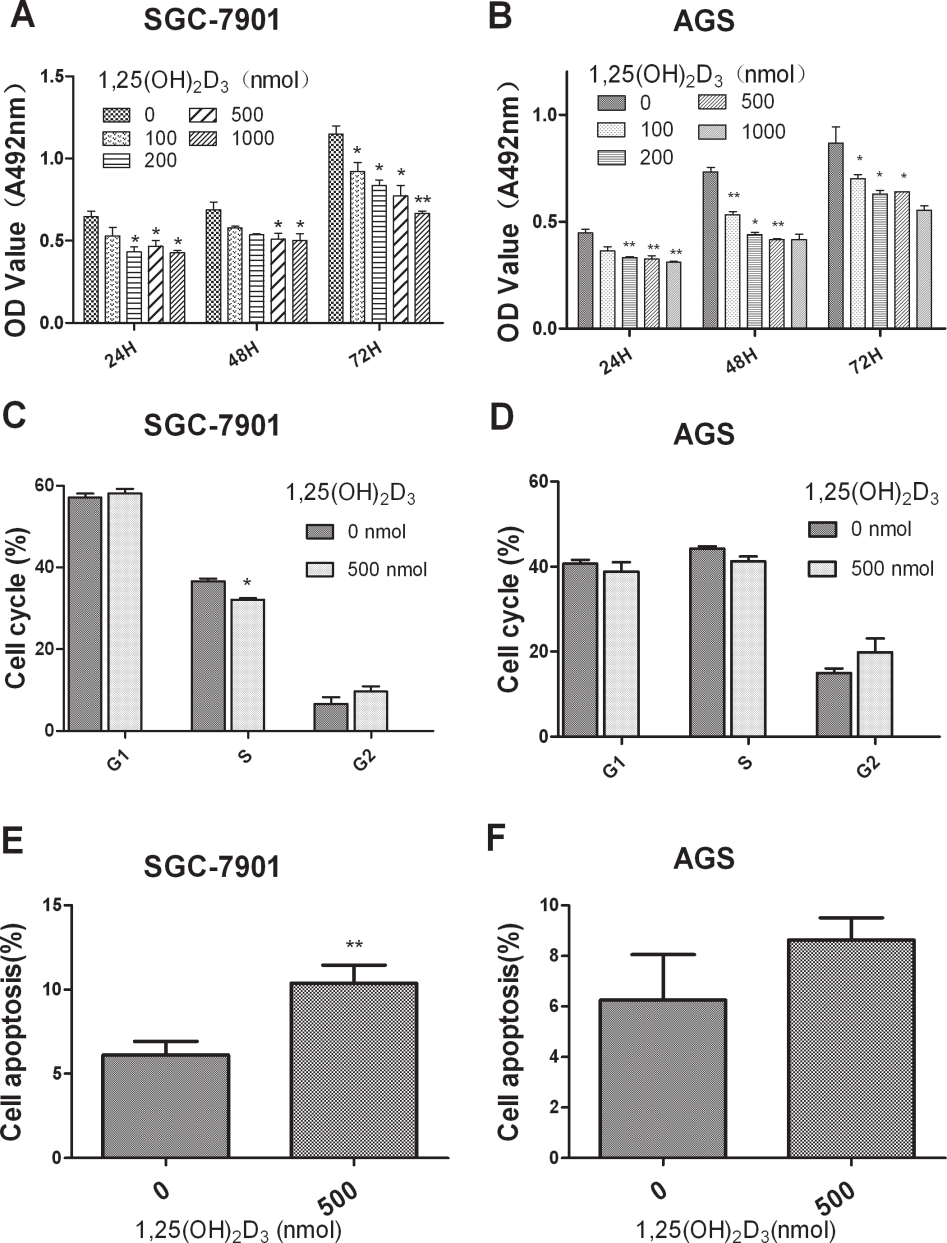
1,25(OH)2D3 inhibits GC cell proliferation and promote cell apoptosis *in vitro* **(A–B)** SGC-7901 and AGS cells were treated with various doses of 1,25(OH)_2_D_3_ and the effects were determined by MTT assay after 24, 48 and 72 hours. **(C–D)** SGC-7901 and AGS cells were treated with 500 nM 1,25(OH)_2_D_3_ for 48 hours and cell cycle distribution was analyzed by flow cytometry. Histogram indicated the percentage of cells in G1, S and G2 cell-cycle phases. **(E–F)** SGC-7901 and AGS cells were treated with 500 nM 1,25(OH)_2_D_3_ for 48 hours, apoptosis was determined by Annexin V staining and flow cytometry. The experiments have been repeated 3 times, representative results of 3 independent experiments were shown. Data shown are mean values. (*P < 0.05; **P < 0.01; ***p < 0.001.)

To examine the effects of 1,25(OH)_2_D_3_ on GC cells, SGC-7901 and AGS cells were treated with various doses of 1,25(OH)_2_D_3_. The MTT assay indicated that 1,25(OH)_2_D_3_ significantly suppressed cell growth and mostly reached a plateau at the 500 nmol dose point when compared with the control group *in vitro* in both cell lines (Figure 1A and 1B). We then studied the potential mechanism of 1,25(OH)_2_D_3_-induced growth suppression. The cells were incubated in serum-free medium to synchronize them in the G1 phase. 1,25(OH)_2_D_3_ slightly decreased the percentage of cells in the S phase in SGC-7901 cells while no obvious change in AGS cells (Figure 1C and 1D). In addition, annexin V staining analysis dispalyed that 1,25(OH)_2_D_3_ promoted cancer cell apoptosis, which is consistent with the study of vitamin D-induced apoptosis through PTEN upregulation [19] (Figure 1E and 1F). Based on these findings, we concluded that 1,25(OH)_2_D_3_ could regulate GC cell proliferation and apoptosis.

### 1,25(OH)_2_D_3_ induces miR-145 expression, which mediates the antitumor activity of 1,25(OH)_2_D_3_

**Figure 2:**
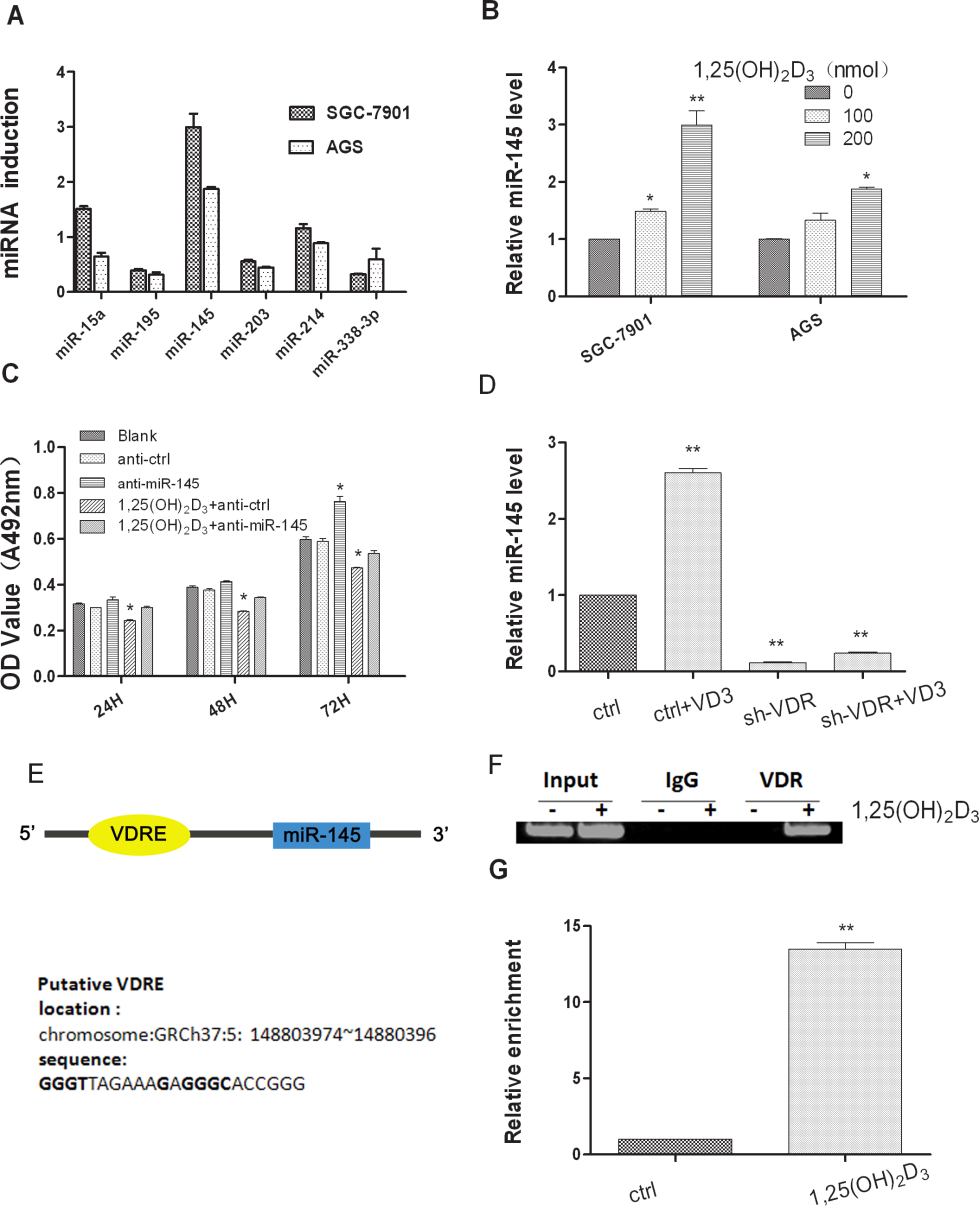
1,25(OH)_2_D_3_ induces miR-145 expression in gastric cancer cells **(A)** SGC-7901 cells and AGS cells were both treated with 200 nM 1,25(OH)_2_D_3_ for 48 hours and total RNA was isolated from the cells and qRT-PCR analysis of miRNAs fold change was performed as described in Materials and Methods. **(B)** qRT-PCR analysis of miR-145 was quantified, and values are expressed as -fold change **(C)** miR-145 inhibitor or ASO-NC transfected SGC-7901 cells were treated with 500 nM calcitriol, Cell growth was determined at 24, 48 and 72 hour time points by MTT assay. **(D)** Empty vector or sh-VDR transfected SGC-7901 cells were treated with 200 nM calcitriol for 24 hours, total RNA was isolated and qRT-PCR analysis was performed. **(E)** Lists of the putative VDRE sequences and the human miR-145 locus in chromosome5. **(F)** The *in vivo* interaction of VDR with miR-145 VDRE was shown. SGC-7901 cells were treated with 500 nM 1,25(OH)_2_D_3_ or blank control for 48 hour, and ChIP assays were performed with control (rat IgG), anti-VDR antibody. **(G)** qRT-PCR analysis was performed with primers spanning predicted VDRE of miR-145. All qRT-PCR results are expressed as mean ± SEM from at least three independent experiments. (*p < 0.05; **p < 0.01.)

To understand the mechanism involved in 1,25(OH)_2_D_3_ cancer growth inhibition, the effects of 1,25(OH)_2_D_3_ on miRNA expression in human GC were analyzed. The expression of several miRNAs in RNA samples extracted from SGC-7901 and AGS cells treated with 0.2 μmol 1,25(OH)_2_D_3_ or blank control was analyzed by quantitative real-time polymerase chain reaction (qRT-PCR) (Figure 2A). Among them, the expression level of miR-145 was significantly increased by three folds (Figure 2B). Therefore we further studied the role of miR-145 in 1,25(OH)_2_D_3_ antitumor activity. To validate the cell function affected by the change of miR-145 expression regulated by 1,25(OH)_2_D_3_, the MTT assay showed that when miR-145 was inhibited, anti-proliferative effect of 1,25(OH)_2_D_3_ decreased (Figure 2C). To determine if VDR was required for miR-145 expression, we transfected a small hairpin RNA against VDR, sh-VDR and a control shRNA into SGC-7901 cells, VDR mRNA and protein expression level were low compared with those of the control shRNA transfected cells (Supplementary Figure 1). As shown in Figure 2D, miR-145 levels were decreased in sh-VDR transfected cells. When sh-VDR transfected cells were treated with 0.2 μmol 1,25(OH)_2_D_3_, miR-145 expression level were rescued, but not totally (Figure 2D). We predicted a candidated VDRE at the upstream of miR-145 locus of human chromosome 5 (named as miR-145-VDRE) by bioinformatics based on the known VDRE motif sequences (Figure 2E). To validate our hypothesis that the VDRE interacts with the VDR *in vivo*, we conducted ChIP assay in SGC-7901 cells. qRT-PCR analysis showed that 1,25(OH)_2_D_3_ induced a significant increase of miR-145-VDRE using DNA purified from ChIP assay (Figure 2G). Antibodies against the VDR precipitated genomic DNA fragments miR-145-VDRE in cells treated with 1,25(OH)_2_D_3_, and no DNA was detected in IgG precipitates (Figure 2F), showing that the recruitments between VDR and the miR-145-VDRE was induced by 1,25(OH)_2_D_3_. Based on these findings, we concluded that miR-145 is induced through VDR and which is critical for 1,25(OH)_2_D_3_ actions.

### miR-145 is frequently downregulated in GC tissues and cell lines

**Figure 3:**
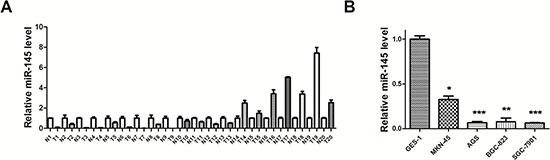
miR-145 is underexpressed in GC tissues and cell lines **(A)** qRT–PCR analysis of miR-145 expression level in human GC tissues (20 paired gastric cancer and adjacent non-tumor tissues). **(B)** qRT–PCR analysis of miR-145 expression level in normal gastric mucosa and GC cells. All qRT-PCR results are expressed as mean ± SEM from at least three independent experiments. (*p < 0.05; **p < 0.01.)

In our previous miRNA microarray analysis, we found that miR-145 was reduced in GC tissues compared with normal gastric tissues [20]. To confirm and extend this finding, we examined the expression of miR-145 in 20 pairs of GC and normal tissues (Supplementary Table 1), and four human gastric cell lines including SGC-7901, AGS, BGC-823, MKN-45 and normal GES-1 by qRT-PCR. miR-145 was significantly downregulated in 15 of 20 (75%) cancer samples (Figure 3A). Additionally, all four gastric cancer cell lines showed > 50% reduction compared with normal cells (Figure 3B). miR-145 reduction suggests that it may act as a tumor suppressor in GC.

### Effects of miR-145 transfection on cell growth and proliferation in GC cell lines

**Figure 4:**
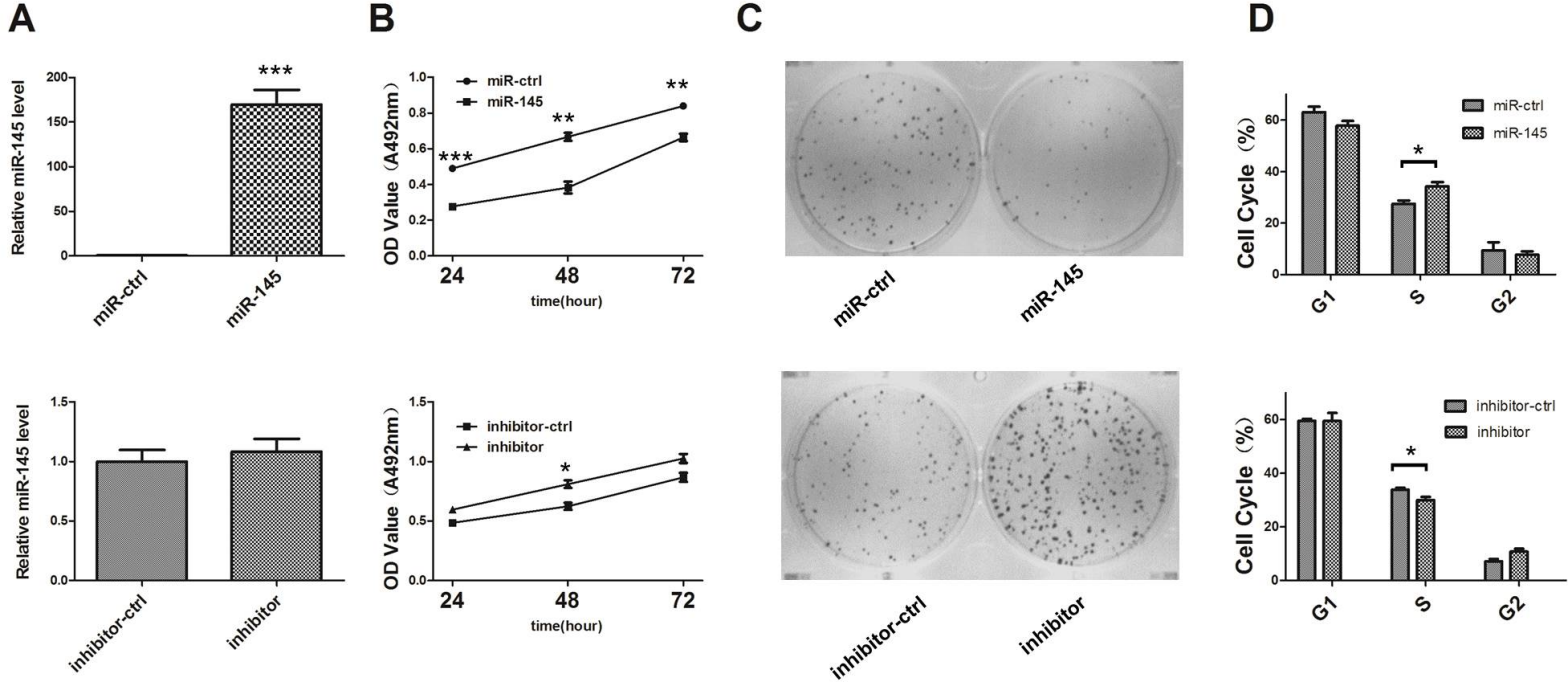
miR-145 inhibits GC SGC-7901 cell growth *in vitro* **(A)** qRT-PCR analysis of miR-145 in SGC-7901 cells transfected with miR-145 over-expression construct or miR-145 inhibitor. All qRT-PCR results are expressed as mean ± SEM from at least three independent experiments. **(B)** The effects of miR-145 on SGC-7901 cell viability were determined by MTT assay at 24, 48 and 72 h after transfection with miR-145 over-expression construct or miR-145 inhibitor, with empty vector or ASO-NC, respectively. **(C)** Representative micrographs of crystal violet-stained cell colonies were analyzed by colony formation assay at day 12 after transfection. **(D)** Histogram indicated the percentage of cells in G1, S and G2 phases after transfection for 48 h based on the flow-cytometric analysis. Data were presented as mean ± SEM. (*p < 0.05; **p < 0.01; ***p < 0.001).

To investigate the functional role of miR-145, we performed gain-of-function and loss-of function studies by transfecting miR-145 expression vector, empty vector, miR-145 inhibitor, and control oligos into the SGC-7901 and AGS cell lines. qRT-PCR was then performed to validate miR-145 expression after transfection. miR-145 expression level was increased 200-fold 48 h after transfection of the miR-145 expression vector and almostly no fold change after miR-145 inhibitor tranefection (Figure 4A). The MTT assay and colony formation assay dispalyed a significant inhibition of cell growth and colony formation after miR-145 transfection when compared with cells transfected with the empty vector (control) in both cell lines, but increased by transfecting miR-145 inhibitor (Figure 4B and 4C; Supplementary Figure 2A–2D). We then study the impact of miR-145 on cell cycle progression. Both cell lines showed overexpression of miR-145 caused the accumulation of cells in the S phase and a corresponding reduction of cells in the G2/M phase, it's consistent there was a decrease percentage of cells in S phase when transfected miR-145 inhibitor (Figure 4D; Supplementary Figure 2E and 2F). These results suggested that miR-145 blocked the S/G2 transition in GC cells *in vitro*.

### E2F3 and CDK6 are both direct targets of miR-145

**Figure 5:**
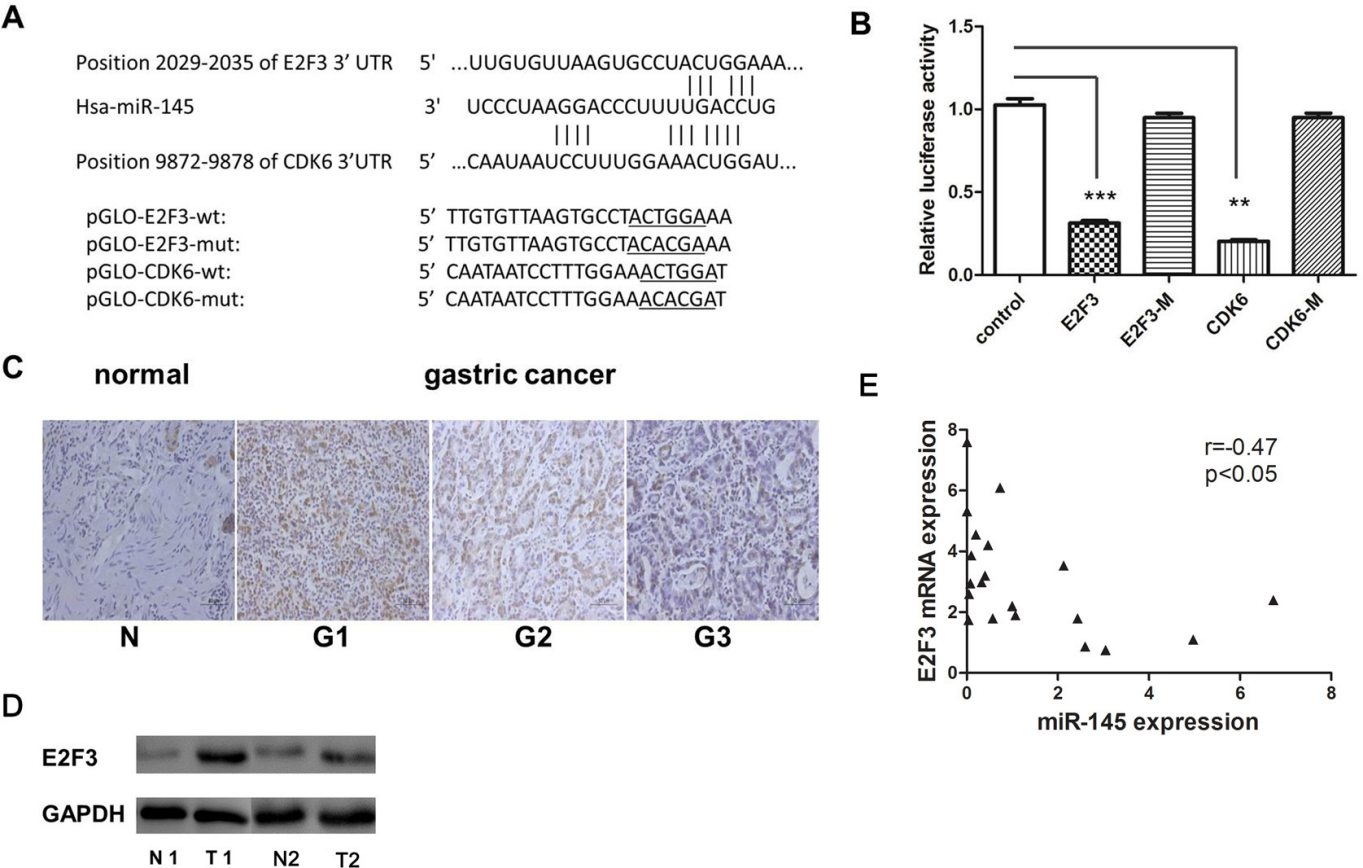
E2F3 and CDK6 are both direct targets of miR-145 **(A)** Scheme of the potential binding sites of miR-145 in the 3′ UTR of E2F3 and CDK6. **(B)** Luciferase assay in SGC-7901 cells. Pre-miR-145 was cotransfected with target gene reporter construct (WT or MUT version of pGLO constructs) or NS-control. Luciferase activity in pGLO-E2F3 and pGLO-CDK6 group displayed a significant decrease following ectopic expression of miR-145. (*p < 0.05; **p < 0.01; ***p < 0.001, Student's *t*-test) **(C)** E2F3 protein expression level measured by IHC in gastric cancer tissue (G1, poorly differentiated;G2 moderately differentiated;G3 well differentiated) **(D)** E2F3 protein level measured by western blotting in GC and adjust normal tissue, 2 paired sample presented. **(E)** Inverse correlation between miR-145 and E2F3 expression in GC tissues. Statistical analysis was performed using Pearson ’ s correlation coefficient (*r* = − 0.47, *P < 0.05).

**Figure 6:**
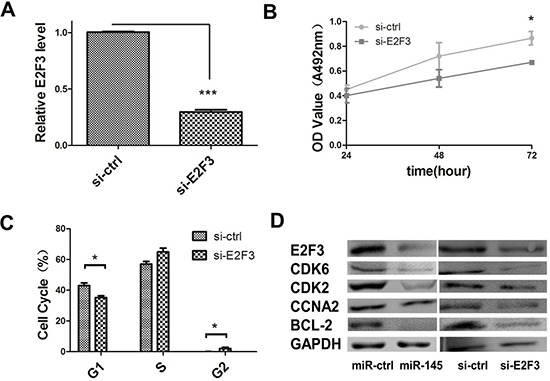
miR-145 inhibits cell proliferation through E2F3 dependent cell cycle regulation **(A)** qRT–PCR were performed to determine the expression level of E2F3 after transfection of si-E2F3 (*P < 0.05, Student's *t*-test). **(B)** MTT assay was performed to determine the growth of SGC-7901 cells treated with si-E2F3. Data are reported as mean ± s.d. for three independent experiments. **(C)** Cell cycle determined in SGC-7901 cells 48 h after transfection of si-E2F3 by propidium-iodide staining flow cytometry. Histogram indicated the percentage of cells in G1, S and G2 cell-cycle phases. **(D)** Protein expression analysis for E2F3 dependent cell cycle regulation proteins in miR-145 over-expression construct, with empty vector orsi-E2F3, si-control-transfected SGC-7901 cells by western blotting.

To understand the mechanism of miR-145-induced inhibition of cell proliferation in GC, miR-145 targets were identified using computer-aided miRNA target prediction programs, such as TargetScan (http://www.targetscan.org/) and other miRBase linked websites (http://www.mirbase.org/). Four putative miR-145 target genes that might play a role in cell proliferation were identified, including *CDK6*, *E2F3*, *CCND2*, and *ERBB4*, as shown in Figure 5A, the binding sites at E2F3 and CDK6 3′-untranslated region (UTR) were displayed. Luciferase reporter assays showed that two of them, *CDK6* and *E2F3*, induced 70% and 60% reduction in luciferase activity compared with vector control, respectively (Figure 5B). We focused on E2F3 because CDK6 has been reported as a target of miR-145 in colon cancer [1]. The miR-145 target sequence in the 3′ - UTR of E2F3 is highly conserved in human and other species. E2F3 is an important cell cycle regulation gene that is highly expressed in GC tissues (Figure 5C and 5D). Next, we determined whether ectopic expression of miR-145 suppressed endogenous E2F3 at the protein level by western blot. As shown in Figure 6D, miR-145 suppressed both *E2F3* and *CDK6*. miR-145 also induced downregulation of *CDK2* and *CCNA2*, which are vital cell cycle regulators (Figure 6D). Interestingly, miR-145 expression level *in vivo* was inversely-correlated with E2F3 mRNA expression level, which was verified by Pearson's correlation coefficient test (Figure 5E). Taken together, our data demonstrated that miR-145 target E2F3 and CDK6 directly and suppress their expression at translation level in SGC-7901 cells.

### Effect of E2F3 knockdown on cell growth and proliferation in the GC cell line SGC-7901

Having validated E2F3 as a direct target of miR-145, we further determined the role of E2F3 in miR-145 mediated suppression of cell proliferation. We next established that the inhibition of cell proliferation involved E2F3 dependent cell cycle regulation. The effect of E2F3 knockdown in SGC-7901 cells was analyzed. si-E2F3 and control siRNA were transfected into SGC-7901 cells. E2F3 mRNA and protein expression levels were both decreased in si-E2F3 transfected cells compared to those of si-control transfected cells (Figure 6A). The MTT assay revealed a significant cellgrowth inhibition of si-E2F3 transfected cells in comparison with that of si-control transfected cells (Figure 6B). Moreover, E2F3 knockdown also induced the accumulation of cells in the S phase, as did miR-145 overexpression (Figure 6C). Consistent with miR-145 ectopic expression, si-E2F3 affected the protein expression level of CDK2 and cyclin A2 (CCNA2) (Figure 6D). These data indicated that miR-145 targeting of E2F3 was responsible for the inhibition of cell proliferation, suggesting a novel therapeutic application for GC treatment.

## DISCUSSION

1,25(OH)_2_D_3_ and vitaminD signaling play a crucial role in inhibiting the development and progression of multiple cancers and miRNAs might be key mediators of 1,25(OH)_2_D_3_ anticancer functions [21, 22]. In our study, we first identified that miR-145 is induced by 1,25(OH)_2_D_3_ and mediatesthe antiproliferative and gene regulatory effects of vitaminD in GC.

Preclinical studies in cells and animal models support the hypothesis that 1,25(OH)_2_D_3_ could inhibit cancer development and progression and circulating vitamin D concentration level has been associated with an increased risk for breast, colorectal and prostate cancer [23–25]. A previous study demonstrated that up to 57.9% patients with GC were deficient in vitamin D and that vitamin D3 can induce apoptosis in GC cells [26]. Some studies suggest that vitamin D may prevent GC from progressing by modulating the extracellular microenvironment. In fact, vitamin D has been shown to alter the expression of multiple genes such as patched1, Gli1, cyclin D1, and Bcl2 in the extracellular matrix remodeling [28]. In our study, we also found that 1,25(OH)_2_D_3_ inhibits cell proliferation and promotes cell apoptosis in two GC cell lines (Figure 1). VDREs have only been identified in the precursor sequences of miR-98 and miR-498 [21, 28] and the expression of both miRNAs is increased via VDRE in prostate and breast cancer. Only few other miRNAs, whose expression is increased or decreased by 1,25(OH)_2_D_3_, but not through VDR, were identified [22, 29, 30]. In our study, among the miRNA analyzed, miR-145 is significantly induced by 1,25(OH)_2_D_3_ in GC cells (Figure 2A). miR-145 expression level was rescued when the sh-VDR transfected cells were treated with 1,25(OH)_2_D_3_, indicating that VDR is required for the activation of miR-145 expression (Figure 2D). Indeed, A VDRE, a cis-acting element at the upstream sequence of miR-145, was identified and verified by CHIP assay (Figure 2F). Thus, miRNA regulation participates and plays an important mediater role in the anticancer actions of 1,25(OH)_2_D_3_.

Deregulation of miRNAs has been reported in many types of human disorders, including cancer. Earlier studies showed that miR-145 is commonly downregulated in cancer and miR-145 overexpression could inhibit not only cell proliferation, but also cell invasion and metastasis by targeting multiple cancer related genes [31–37]. Consistent with these studies, we found that miR-145 is downregulated in GC through our earlier miRNA microarray study [20]. We further confirmed that miR-145 is accurately suppressed in GC tissues and cell lines by qRT-PCR and believed that miR-145 functions as a suppressor in GC. In a series of cell experiments, gain and loss of function studies showed that miR-145 could inhibit cell proliferation by stopping cells in the S phase and blocking the S/G2 transition *in vitro*. Our luciferase assay showed that E2F3 is a direct target of miR-145. CDK6 is also a direct target of miR-145 in GC. Another team confirmed its expression in colon cancer [38]. miR-145 transfection inhibited E2F3 and CDK6 protein expression and E2F3-regulated cell cycle genes such as *CDK2* and *CCNA2* were inhibited. Our results highlight the significance of miR-145 as a tumor suppressor in cell proliferation by targeting E2F3 in GC.

E2F3, a member of E2F family of transcription factors, is known as a potent regulator of the cell cycle and apoptosis with the capacity to stimulate quiescent cells to proliferate or to induce cell apoptosis. Deregulation of E2F3, either overexpression [39] or inactivation by repressor mechanisms [40], is a frequent oncogenic event in human tumorigenesis. The human *E2F3* mRNA contains multiple regulatory features within a long 3′-UTR, including a large number of miRNA seed sequences, two PRE motifs, two NRE motifs, and several alternative polyadenylation signals. It has been shown that some miRNAs that target E2F3 such as 200b,125b, and 503a are downregulated [41, 42]. In our study, we also showed that E2F3 was overexpressed in GC tissues and cell lines. Its expression was inverselycorrelated with that of miR-145. E2F3 inhibition by siRNA had the same effect on GC cell growth than miR-145 overexpression. These results indicate that miR-145 functional roles involve the E2F3 signaling pathway. Together with miR-145, which has multiple target genes involved in cell cyclesuch as *CDK6*, *c-Myc*, and *EGFR*, E2F3 affects a number of genes involved in cell cycle progression, including cyclinE, cyclinA2, *CDC2*, *b-Myb*, and *E2F1*, whose transcription is downregulated in E2f3+/−cell lines that presented no detectable cell cycle defect [43]. Thus, two groups of genes in the cell cycle network are linked by the direct correlation between miR-145 and E2F3. Additionally, miR-145 inhibits E2F3 expression *in vitro*. A study shows that miR-145 could be reversely regulated by E2F3 [44]. It indicates that miR-143/-145 display significantly downregulation in E2F-deficient cells, despite the lack of acute induction in E2F-inducible cell lines. According to the literature, cell cycle arrest may be a prerequisite step for initiating terminal differentiation. Although G1 arrest has been the center of attention in differentiation, some reports indicatedthe involvement of G2 and S-phase arrest. Thus, our results (cells accumulating in S-phase) can explain why some clusters may accumulate in several cell cycle phases, but are not significantly induced during the early exit from quiescence. The question of how E2F3 is overexpressed in GC is still open, but one possible mechanism is through regulation of miR-145 aberrant expression.

**Figure 7:**
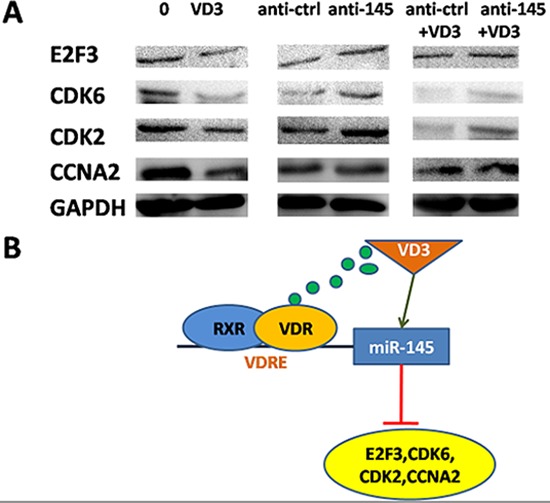
miR-145-mediated calcitriol inhibited expression of cell cycle proteins **(A)** Western blot analyses in SGC-7901 cells after treatment of 1,25(OH)_2_D_3_ or transfection of miR-145 inhibitor. **(B)** Proposed model for miR-145 mediates the antiproliferative and gene regulatory effects of vitamin D3 by directly targeting E2F3 in GC cells.

In summary, we identified miR-145 as a novel target of 1,25(OH)_2_D_3_ in human GC. In addition, E2F3 and CDK6, the direct targets of miR-145, as well as their downstream cell cycle genes (*CDK2* and *CCNA2*) are downregulated by 1,25(OH)_2_D_3_ (Figure 7A). Above all, we provide a novel evidence that 1,25(OH)_2_D_3_ inhibits cell proliferation in human cancer. miR-145, which presents multiple gene regulatory effects in human cancer, was identified as a direct target of 1,25(OH)_2_D_3_. Our results provide new insights on the vitamin D pathway in GC (Figure 7B). A further challenge will be to identify more targets of 1,25(OH)_2_D_3_ and to elucidate its inhibitory effects on GC.

## MATERIALS AND METHODS

### Clinical cases

Paired GC and adjacent non-tumor gastric tissues were collected from patients who had undergone surgical gastric resection at the First Affiliated Hospital of Xi'an Jiaotong University. No local or systemic treatment had been conducted before operation. Tissue samples were immediately snap frozen in liquid nitrogen until RNA extraction. Both tumor and non-tumor tissues were histologically confirmed. Informed consent was obtained from each patient and was approved by the Institute Research Ethics Committee at Cancer Center, Sun Yat-sen University.

### Cell lines and gene transfer

Four human gastric cancer cell lines SGC-7901, AGS, BGC-823, MKN-45 and one common cell line GES-1 were obtained from freeze-stored cells in our Biomedical Reasearch Center, medical school of Xi'an JiaoTong University. All cell lines were maintained in RPIM1640 (Hyclone), containing 10% fetal bovine serum (FBS, PAA Laboratories GmbH, Pasching, Austria) with 100 units/mL penicillin and 100 units/mL streptomycin sulfates and cultured in a humidified 5% CO2 incubator at 37°C. Cell line SGC-7901 was transfected with Lipofectamine 2000 (Invitrogen) following the manufacturer's protocol.

### Plasmid construction and oligonucleotides

pcDNA6.2GW/eGFP was purchased from Invitrogen MiRNA Expression Vector: Synthetic primary transcript of miR-145 (Beijing AuGCT DNA-SYN Biotechnology Co.Ltd) designed into EcoRI and HindIII enzyme sites oligonucleotides were cloned into the pcDNA6.2GW/EmGFP vector and named PcDNA 6.2GW/miR-145. Target gene 3′UTR clone Vector: Synthetic miR-145 target sites (Beijing AuGCT DNA-SYN Biotechnology Co.Ltd) in 3′UTR of target genes were ligated into the pmirGLO Dual-Luciferase (Promega) at the sites of SacI and XhoI. Meanwhile the mutated target 3′UTR vector were also cloned Single oligonucleotides paried with miR-145 regulatory element was synthetized as miR-145 inhibitor. siRNA and shRNA against E2F3 and VDR (GenePharma, Shanghai, China) are perchased. All sequences are shown in Supplementary Table 2.

### RNA extraction, cDNA synthesis and qRT-PCR

Total RNA was extracted from prepared gastric samples with Trizol (Invitrogen, Carlsbad, USA) reagent and cDNA was synthesized according to the manufacturer's protocol (MBI Fermentas). Quantitative RT-PCR was performed using a standard SYBR Green PCR Master Mix (Toyobo, Osaka, Japan), and PCR-specific amplification was conducted in the Applied Biosystems (ABI7500) real-time PCR machine. The relative expression of genes (miR-145, U6, *E2F3, VDR, GAPDH*) was calculated with the 2−(ΔΔCt) method (Livak and Schmittgen 2001). The primers used are listed here (Supplementary Table 3).

### MTT assay

Gastric cancer SGC-7901 cells were seeded into 96-well plates at 5 × 10^3^ cell/well in 200 μl/well culture medium (RPMI1640), and cultured 24 h, 48 h, 72 h after treated or transfected with calcitriol (Cayman) or vector control, miR-145 expression vector, inhibitor control and miR-145 inhibitor. Before the indicated time, 20 μl MTT (Sigma) was added to each well. Then the plates were incubated at 37°C for 4 hour. Cell viability was assayed on FLUO star OPTIMA (BMG). Each experiment contained three replicates and was repeated at least three times.

### Cell cycle assay

Cells were selected from 12-well plates (5 × 10^4^ cells/well) and washed with phosphate-buffered saline (PBS) twice, fixed in 70% ethyl alcohol at 4°C overnight, then washed twice again and added 150 μl 0.1 mg/ml Rnase A and 0.05 mg/ml propidium iodide (PI) each to incubate at 4°C for 30 min. Populations in G0–G1, S, and G2-M phase were measured by flow cytometry with a flow cytometer (FACSort; Becton).

### Dual luciferase reporter assay

SGC-7901 cells were seeded in a 96-well plate (Corning) at a density of 1 × 10^4^ cells per well one day before transfection.miR-145 expression vector was co-transfected with wild or mutated 3′-UTR of CDK6 and E2F3 reporter constructs and a blank pmirGLO Dual-Luciferase as a positive control into cells using Lipofectamine 2000 according to the manufacturer's protocol (Invitrogen). After 24 h, firefly and Renilla luciferase activities were measured using the Dual-Glo luciferase assay system according to the manufacturer's instructions (Promega).

### Western bolt analysis

All gastric cancer cells or tissue were lysedusing RIPA buffer, supplemented with protease inhibitor (invitrogen). Protein concentration was estimated by quantitative analyzer (GeneQuant pro RNA/DNA). Protein was then separated with a 8% to 10% SDS-PAGE (Invitrogen), transferred to a nitrocellulose membrane, incubated with the VDR, E2F3, CDK6 (Abcam, diluted 1/1000), CDK2, CCNA2, BCL-2 (Cell Signaling Technology, diluted 1/1000) and GAPDH (Bioworld, diluted 1/2000) antibodies. After washed three times with TBST, the membrane was incubated with a goat anti-rabbit antibody (Bioworld, diluted 1/3000). Relative protein expression was normalized to GAPDH.

### Immunohistochemistry

Immunohistochemistry (IHC) was performed according to the methods described previously [45]. The sections were pretreated with microwave, blocked, and incubated using polyclonal rabbit anti-human E2F3 (Abcam, USA). Staining intensity was assessed.

### Chromatin immunoprecipitation assay

For chromatin immunoprecipitation (ChIP) assays, SGC-7901 cells were treated with 500 nM 1,25(OH)_2_D_3_ (Cayman, USA) for 48 hours and cross-linked with formaldehyde (endconcentration = 1%) for 15 min at room temperature, and the reactions were quenched with glycine (0.125M) for 30 min. Rinse the cells twice with 5 mL 1x PBS. Harvest cells and then nuclei were resuspended by Mg-NI, Mg-NI-XP40, Ca-NI (0.5M EGTA additon) and lysis buffer (Protease inhibitor stock addtion) in turn. Then a sonicator was used to shear the cross-linked DNA to an average length of 100 to 500 bp and centrifuged at 14,000 rpm to remove insoluble material. Sheared chromatin was immunoprecipitated with 1 μg of anti-VDR or IgG antibody (Abcam, USA) overnight at 4°C. The immunocomplexes were collected with 40 μl Protein G agarose (Invitrogen, USA) and slurry for 2 hour at 4°C. The beads were washed and reverse the cross-links with proteinase K by incubating at 65°C for 8 hour. DNA was purified by phenol/chloroform (Invitrogen, USA) extraction and ethanol precipitation and used as the template for quantitative real-time PCR with the primer sets for ChIP-PCR assay (Supplementary Table 3).

### Statistical analysis

Statistical analysis was performed according to the methods described previously [45].

## SUPPLEMENTARY FIGURES AND TABLES


