# Supplementary material for: miR-145 mediates the antiproliferative and gene regulatory effects of vitamin D3 by directly targeting E2F3 in gastric cancer cells
Source: Oncotarget. 2015 Feb 18;6(10):7675–85. doi: 10.18632/oncotarget.3048 (PMC4480708; doi:10.18632/oncotarget.3048)
Supplement: Supplementary file 1 [file oncotarget-06-7675-s001.pdf]

SUPPLEMENTARY FIGURES AND TABLES

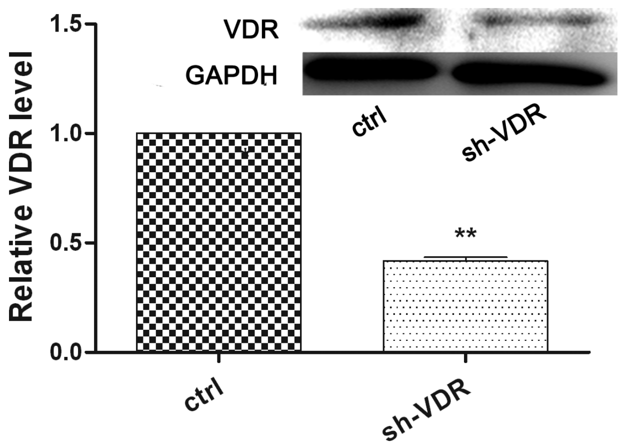

Supplementary Figure 1: qRT–PCR and western blot were performed to determine the expression level of VDR after transfection of sh-VDR.

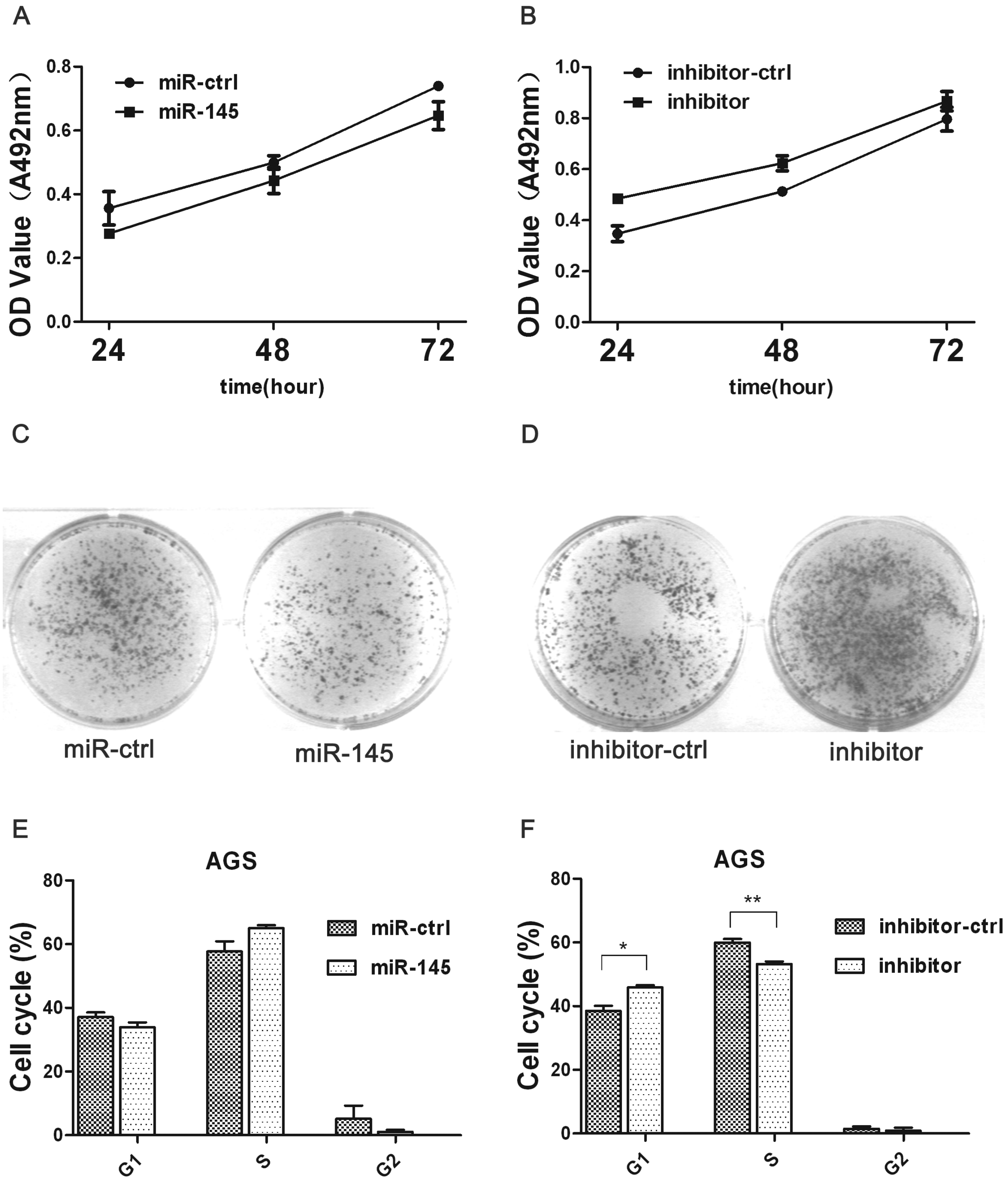

Supplementary Figure 2: miR-145 inhibits GC AGS cell growth *in vitro*. (A–B) The effects of miR-145 on AGS cell viability were determined by MTT assay at 24, 48 and 72 h after transfection with miR-145 over-expression construct or miR-145 inhibitor, with empty vector or ASO-NC, respectively. **(C–D)** Representative micrographs of crystal violet-stained cell colonies were analyzed by colony formation assay at day 12 after transfection. **(E–F)** Histogram indicated the percentage of cells in G1, S and G2 phases after transfection for 48 h based on the flow-cytometric analysis. Data were presented as mean ± SEM. (*$p < 0.05$; **$p < 0.01$).

Supplementary Table 1: The basic information of all patients

Patient No.	Sex	Age	T stage	Histological grade
1	M	69	T4N2M0	IV
2	M	69	TxN1M1	IV
3	F	49	T3N2M1	IV
4	M	48	T4N3M0	IV
5	M	74	T3N2M1	IV
6	M	62	T4N1M0	IV
7	M	62	T2N1M0	IIb
8	M	63	T3N1M0	IIIa
9	M	59	T4N3M0	II–III
10	M	79	T4N1M0	III
11	M	40	T4N1M0	III
12	M	68	T4N1M0	IIIB
13	M	62	T2N2M0	II
14	M	69	T2N0M0	Ib
15	M	45	T1N0M0	I
16	M	63	T4N1M0	IIIa
17	M	59	T4N3M0	IIIC
18	M	66	T2N1M0	IIa
19	M	59	T4N1M0	II
20	M	66	T4N1M0	III

Supplementary Table 2: Oligonucleotides and sh-RNA target sequences in this work

Gene name	Gen bank	Sense oligo sequences (5′-3′)
Hsa-miR-145	NR_029686g	AATTCCACCTTGTCCTCACGGTCCAGTTTTCCC<br>AGGAATCCCTTAGATGCTAAGATGGGGATTCCT<br>GGAAATACTGTTCTTGAGGTCATGGTT
miR-145 inhibitor	NR_029686	GGAGTCCCTCATAACGGTCCTTAGGGA
E2F3–3′UTR-WT	NM_001949	CTTGTGTTAAGTGCCTACTGGAAAC
E2F3–3′UTR-MT	NM_001949	CTTGTGTTAAGTGCCTACACGAAAC
CDK6–3′UTR-WT	NM_001259	CCAATAATCCTTTGGAAACTGGATC
CDK6–3′UTR-MT	NM_001259	CCAATAATCCTTTGGAAACACGATC
Si-E2F3	NM_001949	GACUUCAUGUGUAGUUGAUU
Si-ctrl		UUCUCCGAACGUGUCACGUUU
Sh-VDR	NM_000376	CATCATGTTGCGCTCCAAT

Supplementary Table 3: Primers used in this work

Gene name	Sense primer (5′-3′)	Anti-sense primer (5′-3′)
Hsa-miR-145	CAGTGCGTGTCGTGGAGT	AGGTCCAGTTTTCCCAGG
U6	GCTTCGGCAGCACATATACTAAAAT	CGCTTCACGAATTTGCGTGTCAT
E2F3	ATATCCCTAAACCCGCTTCC	TGGTCCTCAGTCTGTAAGA
CDK6	TGATCAACTAGGAAAAATCTTGGAC	GGCAACATCTCTAGGCCAGT
VDR	GAAGCTGAACTTGCATGAGGA	GTCCTGGATGGCCTCAATC
GAPDH	GCCAAAAGGGTCATCATCTC	GTAGAGGCAGGGATGATGTTC
miR-145-chip	ACCCCCAAGCGGTGTCTCTCATCCT	tacacttcctccgtccccaaccctg
